# Reliability and device objectivity in oscillatory blood pressure measurement – a measurement error analysis to inform clinical decision making

**DOI:** 10.1186/s12872-026-06262-1

**Published:** 2026-07-14

**Authors:** Konstantin Warneke, Niklas Lebelt, Franz Liebermann, Benjamin Jöst, Marco Herbsleb

**Affiliations:** 1 Institute of Sustainability Psychology (ISP), Leuphana University Lüneburg, Lüneburg, Germany; 2 Department for Human Movement Science and Exercise Physiology, Friedrich Schiller University Jena, Jena, Germany; 3 Department of Sports Medicine and Health Promotion, Friedrich Schiller University Jena, Jena, Germany; 4 Department of Psychosomatic Medicine, University Hospital Jena, Friedrich Schiller University Jena, Jena, Germany

**Keywords:** Hypertension; standardized measurement, Systolic blood pressure, Non-invasive blood pressure

## Abstract

The implementation of different hypertension thresholds (e.g. ESC/ESH: 140:90 or ACC/AHH: 130:80) substantially increased hypertension prevalence donation of pharmacological treatment across various populations. Under narrow margins of only 10mmHg, measurement accuracy and reliability become decisive for clinical decision-making. However, detailed information on systematic and random errors naturally occurring in clinical practice under repeated testing conditions remain widely limited.

To provide practically relevant insights, 65 participants were recruited for visited the lab four times with one un-standardized arrival measurement to mimic oftentimes observed clinical real-world scenarios followed by four standardized measurements performed in adherence with current guidelines. Tests were performed with two certified blood pressure devices, each on two testing days to quantify intra- and interday, as well as interdevice measurement errors via relative reliability and systematic and random measurement errors.

Insufficient pre-measurement rest periods caused clinically relevant systematic blood pressure enhancements. Within a subsequent 10–18 min rest, the blood pressure was reliably reduced with about 10 mmHg on both days and both devices. However, the Boso Medicus testing device showed consistently higher blood pressure values with 13mmHg compared to the Welch Allyn monitor.

Although measurements could be replicated inter-day in both devices, substantial interdevice discrepancies and clinically relevant random errors were observed, that can cause false-positive hypertension diagnoses, especially when pre-measurement rest was insufficient. Results underscore the relevance of performing several blood pressure assessments (per day and on different days) to account for random measurement errors and reduce the risk of inappropriate clinical decision-making and unnecessary treatment escalation.

**Supplementary Information:**

The online version contains supplementary material available at 10.1186/s12872-026-06262-1.

## Introduction

Hypertension is one of the most prevalent cardiovascular risk factors worldwide and represents a major contributor to global morbidity and mortality [1–3]. According to the World Health Organization, approximately 1.4 billion adults aged 30 to 79 years are affected, with a substantial proportion remaining undiagnosed [4]. The clinical relevance of elevated blood pressure is undisputed, as even moderate increases are associated with a markedly higher risk of cardiovascular disease, stroke, and premature mortality [5]. Clinical decisions regarding the diagnosis and treatment of hypertension are predominantly based on numerical blood pressure values obtained during routine measurements. Consequently, relatively small differences in measured blood pressure may lead to fundamentally different diagnostic classifications and therapeutic consequences [6]. This issue becomes particularly evident, while divergent international guidelines ranging from diagnostic thresholds between 130/80 mmHg (American College of Cardiology and the American Heart Association) to 140/90 mmHg (European Society of Cardiology and the European Society of Hypertension) [7, 8]. The adoption of these differing thresholds has been shown to substantially increase hypertension prevalence and the number of individuals eligible for pharmacological treatment across various populations [9–13]. Under such narrow margins, measurement accuracy and reliability become decisive for clinical decision-making.

Blood pressure is not a static physiological parameter. Short-term biological variability, daily fluctuations, and situational influences are inherent characteristics of blood pressure regulation [14]. In addition to this intrinsic variability, numerous sources of measurement error have been identified that may systematically or randomly distort blood pressure readings. These include insufficient rest before measurement (+ 4.2–11.6mmHg systolic blood pressure) [15, 16], inappropriate cuff size or placement (smaller cuff: +2.08–11.2mmHg systolic blood pressure) [17, 18], body posture (+ 1.2–10.7 mmHg systolic blood pressure) [19, 20] and environmental factors like the white-coat effect (-7.6–13.3 mmHG systolic blood pressure) [21, 22]. Such deviations may result in systematic over- or underestimation of blood pressure values, thereby compromising internal validity and increasing the risk of diagnostic misclassification [23–25].

The clinical implications of measurement error are substantial. Sakhuja et al. [26] demonstrated that a combination of random and systematic errors in systolic blood pressure measurement can result in misclassification rates of up to 20%. When diagnostic thresholds differ by only 10 mmHg, as is the case between European and American guidelines, even small measurement deviations may determine whether an individual is classified as hypertensive or normotensive and whether pharmacological treatment is initiated. Accordingly, high levels of measurement standardization, the use of validated devices, repeated measurements, and strict adherence to measurement guidelines are strongly recommended to minimize avoidable sources of error [27–31].

Accordingly, the accurate and unbiased determination of blood pressure is considered an essential clinical skill worldwide [2, 32, 33]. However, studies have shown a dearth of knowledge and good practice, calling for further studies to outline potential caveats that must be avoided [33]. In contemporary clinical practice, automated oscillometric blood pressure devices have largely replaced auscultatory measurements. These devices are widely regarded as less dependent on observer expertise, less susceptible to observer bias, and more suitable for standardized and home-based measurements [34, 35]. While validation studies have demonstrated acceptable agreement with reference methods, fewer studies have comprehensively examined the reliability of oscillometric blood pressure measurements under repeated testing conditions. In particular, data on intraday and interday reliability, the magnitude of random and systematic measurement error remain scarce.

In addition to temporal stability, device objectivity represents another source for measurement errors. Due to difference algorithms when calculating the blood pressure, a detailed measurement error analysis might not just be informative in validation studies (oscillatory measurement against gold standard methods) but is also indicated to identify discrepancies between different devices used in the daily practice.

While several sources of blood pressure measurement variability have been described previously, comparatively little evidence integrates intraday and interday reliability, systematic and random measurement error, and interdevice objectivity of oscillometric blood pressure measurements within a unified measurement-error framework under clinically standardized yet practice-relevant conditions.

## Methods

The study was designed as a measurement error analysis to investigate the reliability and objectivity of oscillometric blood pressure measurements under conditions of high procedural standardization. To address these objectives, repeated blood pressure measurements were performed within and across four laboratory visits using two validated oscillometric devices to quantify (a) intraday reliability, (b) interday reliability each with associated systematic and random measurement error, and (c) device objectivity between two certified oscillometric blood pressure monitors. To provide clinically relevant insight, both relative and absolute reliability metrics were examined. Relative reliability (e.g., intraclass correlation coefficient) describes the ability to distinguish between individuals but do not quantify the measurement error [36]. In contrast absolute reliability metrics, including the standard error of measurement [37] and the minimal detectable change [38], quantify measurement error and whether the observed differences exceed random variation to consider clinical relevance [24, 25]. The study design allowed for the separate evaluation of temporal stability (within-session and between-session) and device-related differences while minimizing confounding influences due to biological variability and measurement conditions.

### Participants

A priori sample size estimation using conventional power analysis was not applicable, as reliability and measurement error analyses are not based on hypothesis testing of mean differences. Instead, adequate sample sizes are required to ensure stable estimates of reliability coefficients and error metrics [39]. Previous methodological recommendations suggest a minimum of 30 participants for reliable intraclass correlation coefficient (ICC) estimation [40] and at least 50 participants for robust quantification of random measurement error [24, 41].

Accordingly, sixty-five (*n* = 65) young and healthy adults were recruited from the local University Campus. The overall sample comprised 37 men and 28 women (mean age = 25.0 ± 8.5 years; height = 176.8 ± 10.5 cm; body mass = 72.8 ± 12.5 kg, BMI = 23.3 kg/m^2^ ± 4.9 kg/m^2^). Participants were eligible for inclusion if they were without cardiovascular disease, were not obese, and did not present conditions that could interfere with standardized blood pressure measurement procedures [26, 42].

Participants were blinded to the specific study objectives to simulate routine blood pressure measurements as closely as possible. Written informed consent was obtained from all participants prior to data collection. The study protocol was approved by the local ethics committee (ethics approval number: FSV 25/043) and was performed in adherence with the Declaration of Helsinki. In addition, the study was pre-registered through clinical trial registration (DRKS00040027).

### Measurement protocol

Each participant attended the laboratory on four separate days. At each visit, blood pressure was measured five times following a standardized protocol aligned with national and international guidelines [32, 43].

### Blood pressure evaluation

Upon arrival at the laboratory, an initial blood pressure measurement was performed without a preceding standardized rest period (Baseline). To simulate a real-world scenario, baseline measurements were obtained without standardization and no standardized sitting time (1–3 min), which was required for cuff placement, arm circumference assessment, and device preparation. This procedure was chosen to reflect commonly observed clinical practice, where blood pressure is often measured shortly after patient arrival [44]. Subsequently, participants rested in a seated position for ten minutes, after which four additional blood pressure measurements were obtained (T1–T4), each separated by a three-minute rest interval. One laboratory visit lasted approximately 25 min, of which about 20 min were dedicated to resting periods.

The blood pressure measurement was conducted in adherence with the ESH guideline from Mancia et al. [43]. In detail, participants were instructed to sit comfortably with their back supported, feet flat on the floor, and knee joints flexed at approximaltely 90°. The left arm was supported at heart level and blood pressure measurements were consistently performed on the left arm to reflect clinical screening procedures and to avoid possible side-depending variations. Participants were asked to refrain from caffeine, nicotine, meals, and strenuous physical activity [45] for at least 30 min prior to each visit. Urge to urinate was avoided, and restroom facilities were provided bevor testing. Cuff size was determined prior to the first visit by measuring arm circumference at the midpoint between the dorsal angle of the acromion and the olecranon. Appropriate cuff sizes were selected according to manufacturer specifications. Once positioned, the cuff remained on the arm throughout each session to minimize variability due to repositioning. Cuff placement followed device-specific guidelines over the brachial artery, approximately 2–3 cm above the elbow crease with 2 fingers fitting between the arm and the cuff [45].

**Fig. 1:**
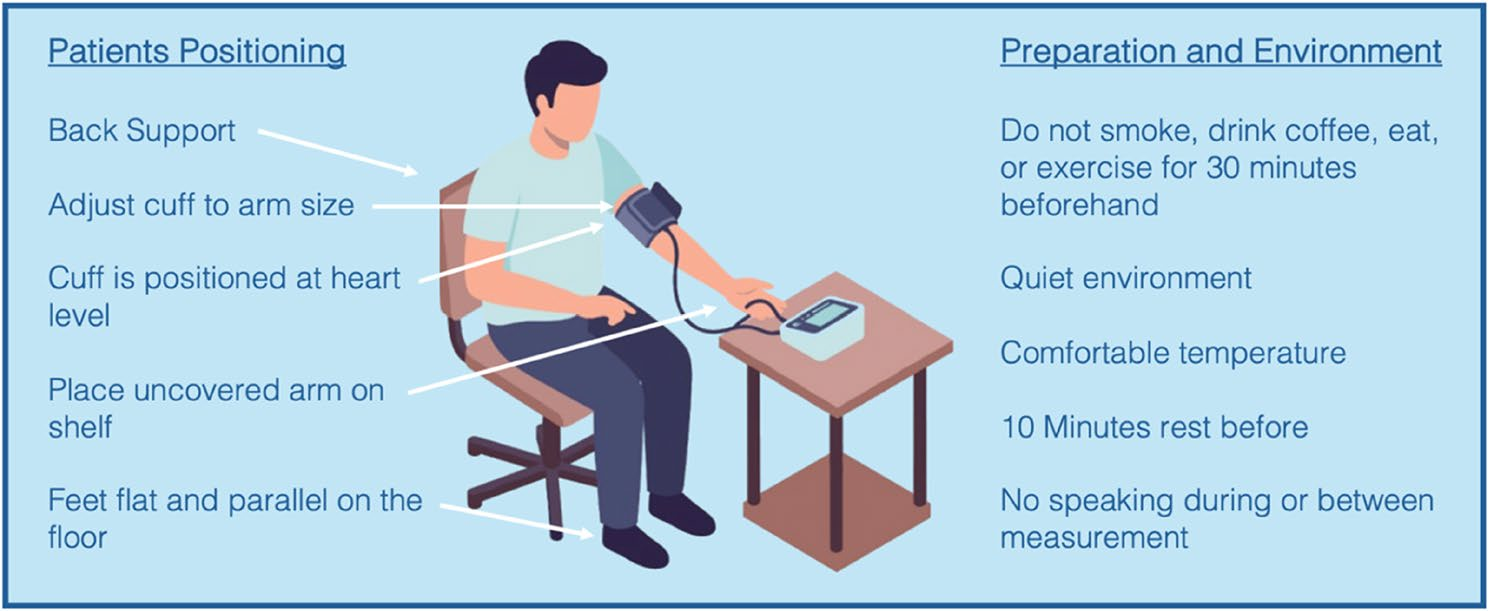
Summarizes the standardized participant positioning and preparation of the environment for the blood pressure measurement in adherences with current guidelines (ESH Guidelines for the management of arterial hypertension)

**Fig. 2:**
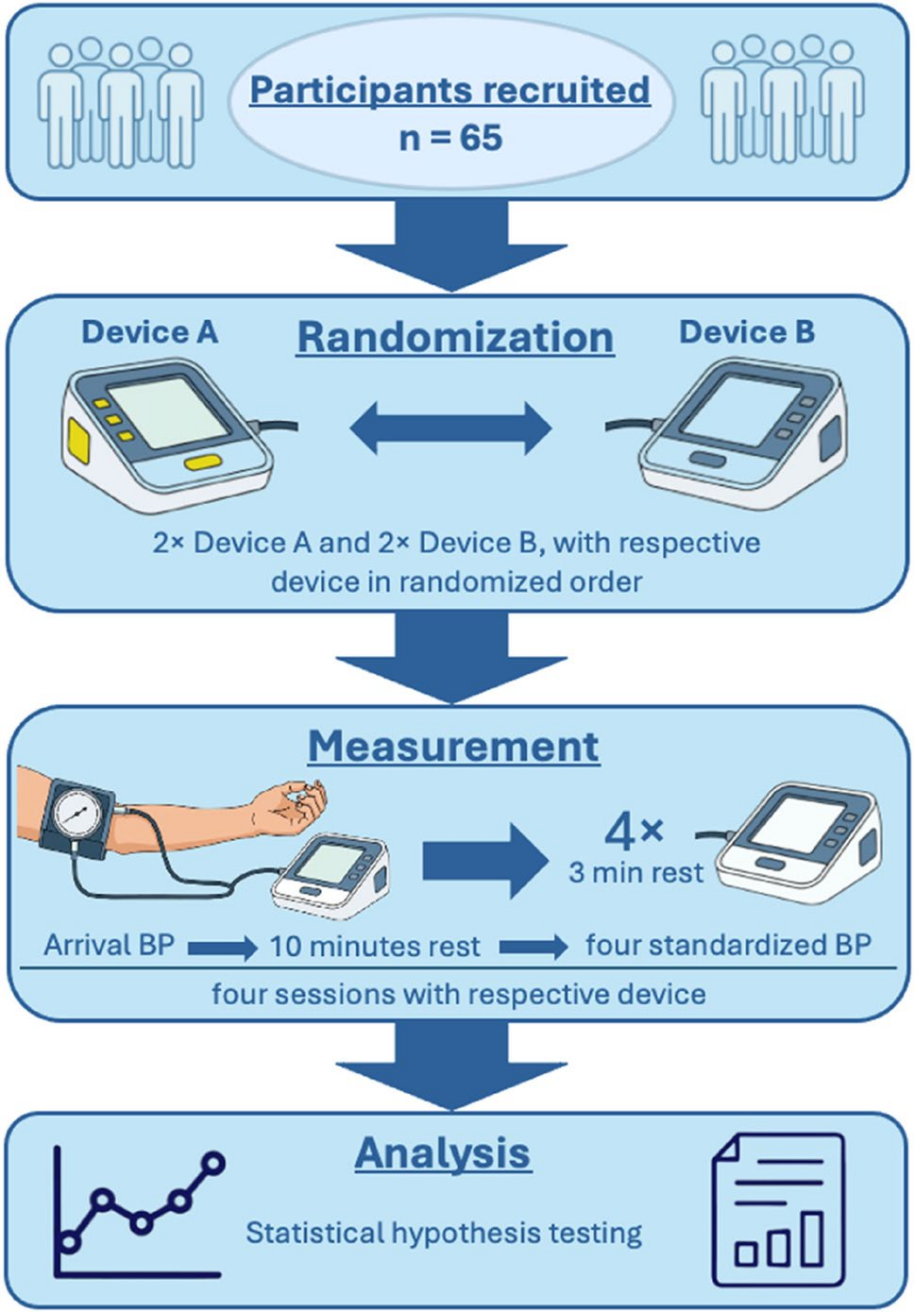
Shows the measurement protocol for determining the reliability and device objectivity of the measuring instruments

The standardized measurement environment and participant positioning are illustrated in Fig. 1, and the overall measurement protocol is summarized in Fig. 2.

#### Blood pressure devices

Two validated and certified oscillometric blood pressure monitors were used: BOSO medicus vital (BOSO GmbH, Jungingen, Germany; cuff size CA04: 22–42 cm) and Welch Allyn ProBP 2000 (Welch Allyn Inc., Skaneateles Falls, NY, USA, cuff sizes: Small Adult 10: 20–26 cm, Adult 11: 25–34 cm, Adult 12: 32–43 cm [46]). Device order was randomized a priori to minimize potential order effects. A randomization list was generated prior to data collection, allocating participants to alternating device sequences across visits.

As only one device could be applied at a time on the same arm, measurements were conducted sequentially according to the randomized order. Both devices were used in accordance with manufacturer instructions prior to and throughout data collection.

#### Outcomes

Systolic and diastolic blood pressure were defined as the primary outcome variables. Heart rate, as provided by the oscillometric devices, was recorded as a secondary outcome to evaluate whether reliability patterns observed for blood pressure were also evident for another physiological parameter.

### Statistic

Statistical analyses were performed unsing JASP (Version 0.19.3, Netherlands, 2025). Descriptive statistics are reported as means (M) and standard deviations (SD). Normality of distributions was assessed using the Shapiro Wilk Test (*p* > 0.05). Given the sample size (*n* > 30), parametric analyses were considered robust against moderate violations of normality.

Intraday systematic differences across the five measurement time points (Baseline, T1–T4) were evaluated using one-way repeated-measures analyses of variance (ANOVA) with Scheffé post hoc tests. Effect sizes were reported as partial eta squared (ηp²), classified as small (≤ 0.06), moderate (> 0.06–0.14), or large (> 0.14). Post hoc effects were quantified using Cohen’s d which was classified as follows: d = 0–0.2 as trivial, d > 0.2 – <0.5 as small, d ≥ 0.5 – <0.8 as moderate and d > 0.8 as large effects [47].

Relative reliability was assessed using intraclass correlation coefficients for absolute agreement (ICC 3,k).

$$\:\mathrm{I}\mathrm{C}\mathrm{C}=\:\frac{{\mathrm{M}\mathrm{S}}_{\mathrm{R}}-\:{\mathrm{M}\mathrm{S}}_{\mathrm{E}}}{{\mathrm{M}\mathrm{S}}_{\mathrm{R}\:}\frac{{\mathrm{M}\mathrm{S}}_{\mathrm{C}}-\:{\mathrm{M}\mathrm{S}}_{\mathrm{E}}}{\mathrm{n}}}$$

Where ICC stands for intraclass correlation coefficient, MS_R_ codes mean square of rows, MS_E_ stands for Mean Square of Error and MS_C_ means Mean Square of Columns. N stands for number.

Interpretation was adopted from Koo & Li [40] classifying an ICC ≥ 0.5–0.75 as moderate, > 0.75–0.9 as good and ≥ 0.9 as excellent reliability. Results were interpreted considering the 95% confidence intervals (95% CI). Using the ICC, the Standard Error of Measurement (SEM) [25] was calculated as a measure of absolute reliability, using:

$$\:\mathrm{S}\mathrm{E}\mathrm{M}=\mathrm{S}\mathrm{D}\mathrm{*}\:\sqrt{1-\mathrm{I}\mathrm{C}\mathrm{C}}$$

Where SEM = standard error of measurement, SD = standard deviation and ICC = intraclass correlation coefficient. In turn, the SEM was used to evaluate the Minimal Detectable Change (MDC) as a measure for practically relevant interventional changes that surpass the sole measurement error, using

$$\:\mathrm{M}\mathrm{D}\mathrm{C}=\mathrm{S}\mathrm{E}\mathrm{M}\mathrm{*}1.96\mathrm{*}\sqrt{2}$$

Where SEM stands for standard error of measurement and MDC was the minimal detectable change.

Random error was further quantified using the Mean Absolute Error (MAE) and the Mean Absolute Percentage Error (MAPE) to facilitate clinical interpretation [48], using the following formula:

$$\:\mathrm{M}\mathrm{A}\mathrm{E}=\:\frac{1}{\mathrm{n}}\mathrm{*}\:\sum\limits_{\mathrm{i}=1}^{\mathrm{n}}\mid\:{\mathrm{x}}_{\mathrm{i}}-\:{\mathrm{y}}_{\mathrm{i}}\mid\:$$

Where MAE stands for mean absolute error, n for number of participants or trials and x_i_ is the first data column and y_i_ the comparator. The MAPE was calculated in adherence with the adjusted version from Warneke et al. [48], using:

$$\:{\mathrm{M}\mathrm{A}\mathrm{P}\mathrm{E}}_{adj}=\:\frac{1}{\mathrm{n}}\mathrm{*}\:\sum\limits_{\mathrm{i}=1}^{\mathrm{n}}\mid\:\frac{{\mathrm{x}}_{\mathrm{i}}-\:{\mathrm{y}}_{\mathrm{i}}}{\left(\frac{{\mathrm{x}}_{\mathrm{i}}-\:{\mathrm{y}}_{\mathrm{i}}}{2}\right)}\mid\:\mathrm{*}100$$

Where MAPE = mean absolute percentage error, n stands for number of participants or trials and x_i_ is the first data column and y_i_ the comparator.

Agreement between repeated measurements and between devices was visually examined using Bland–Altman plots [41].

For interday reliability analyses, mean values of T1–T4 were calculated, as no systematic differences were observed within this resting condition. Device objectivity was evaluated using paired comparisons between devices for baseline and resting conditions. In cases of multiple testing, the family-wise error rate was controlled to reduce the risk of type I error [49]. The level of statistical significance was set at *p* = 0.05.

## Results

All 65 participants completed all four laboratory visits, resulting in a complete dataset without dropouts. Across most measurement sessions, systolic and diastolic blood pressure values normal distribution was assumed unviolated with *p* > 0.05 in most test session. However, some individual measures revealed *p* < 0.05 (e.g. systolic blood pressure for baseline, T3, T4 in day one for the Boso Medicus monitor or on day one Welch Allyn measurements for baseline, T1 to T3). Given the sample size (*n* > 30), parametric analyses were considered robust. To ensure consistency, non-parametric alternatives were calculated for key analyses and yielded comparable results. Median values and interquartile ranges are provided in the Supplemental Material (Table S1).

Results are presented separately for intraday reliability, interday reliability, and device objectivity. Unless otherwise stated, systolic and diastolic blood pressure represent the primary outcomes, while heart rate is reported as a secondary outcome.

### Intraday systematic differences across repeated measurements

Intraday analyses focused on systematic changes across the five repeated measurements obtained within each laboratory visit (Baseline, T1–T4). Comparisons were performed separately for each device (Table 1).

**Table 1:** Intrasession reliability stratified for measurement device and testing day comprising descriptives, relative and absolute reliability as well as systematic and random measurement evaulation

		Parameter	M±SD	M±SD	ICC (95% CI)	SEM	MDC	MAE (MAPE)	Mean diff.	ANOVA
Welch Allyn	SBP_d1 (in mmHg)	Baseline – T1	117.63±11.51	112.94±10.80	0.91 (0.90–0.92)	1.37	3.81	6.48 (5.60)	4.69	*p* = 0.003,$\eta_p{^2}$=0.050
		T1 – T2	112.94±10.80	111.97±10.69	0.93 (0.92–0.94)	0.73	2.01	3.89 (3.46)	0.97	
		T2 – T3	111.97±10.69	111.37±10.78	0.97 (0.96–0.97)	0.39	1.08	3.15 (2.81)	0.60	
		T3 – T4	111.37±10.78	111.05±9.93	0.95 (0.94–0.95)	0.54	1.49	3.40 (3.05)	0.32	
		Baseline – T4	117.63±11.51	111.05±9.93	0.86 (0.84–0.87)	2.17	6.02	8.22 (7.13)	6.59	
	DBP_d1 (in mmHg)	Baseline – T1	72.97±5.72	70.31±6.09	0.86 (0.84–0.88)	1.01	2.81	3.83 (5.32)	2.66	*p* = 0.002,$\eta_p{^2}$=0.051
		T1 – T2	70.31±6.09	69.65±5.93	0.87 (0.86–0.89)	0.73	2.03	2.88 (4.16)	0.66	
		T2 – T3	69.65±5.93	69.03±6.57	0.95 (0.94–0.95)	0.35	0.97	2.21 (3.20)	0.62	
		T3 – T4	69.03±6.57	69.75±5.61	0.91 (0.90–0.92)	0.59	1.64	2.79 (4.03)	-0.72	
		Baseline – T4	72.97±5.72	69.75±5.61	0.86 (0.84–0.88)	1.04	2.88	3.92 (5.51)	3.22	
	HR_d1 (in BPM)	Baseline – T1	71.56±13.12	69.74±11.46	0.95 (0.94–0.95)	0.73	2.04	4.65 (6.59)	1.85	*p* = 0.931,$\eta_p{^2}$=0.003
		T1 – T2	69.74±11.46	70.12±11.15	0.94 (0.94–0.95)	0.73	2.02	4.20 (6.07)	-0.39	
		T2 – T3	70.12±11.15	70.45±13.35	0.86 (0.84–0.88)	1.18	3.26	4.45 (6.04)	-0.32	
		T3 – T4	70.45±13.35	70.75±11.94	0.90 (0.89–0.91)	0.88	2.44	3.94 (5.48)	-0.31	
		Baseline – T4	71.56±13.12	70.75±11.94	0.91 (0.90–0.92)	1.16	3.20	5.45 (7.74)	0.83	
	SBP_d2 (in mmHg)	Baseline – T1	118.60±11.10	112.95±11.70	0.90 (0.89–0.91)	1.55	4.31	6.95 (6.01)	5.65	*P* < 0.001,$\eta_p{^2}$=0.058
		T1 – T2	112.95±11.70	112.95±11.70	0.91 (0.90–0.92)	1.00	2.78	4.73 (4.14)	1.84	
		T2 – T3	112.95±11.70	111.40±9.22	0.93 (0.92–0.94)	0.72	2.00	3.87 (3.50)	-0.26	
		T3 – T4	111.40±9.22	111.57±9.43	0.92 (0.91–0.93)	0.73	2.03	3.67 (3.27)	-0.18	
		Baseline – T4	118.60±11.10	111.57±9.43	0.88 (0.87–0.89)	2.00	5.55	8.17 (7.01)	7.03	
	DBP_d2 (in mmHg)	Baseline – T1	72.57±6.31	69.62±6.87	0.89 (0.87–0.90)	0.90	2.50	3.84 (5.45)	2.95	*p* = 0.002,$\eta_p{^2}$=0.051
		T1 – T2	69.62±6.87	68.76±6.30	0.90 (0.89–0.91)	0.71	1.96	3.17 (4.53)	0.86	
		T2 – T3	68.76±6.30	69.16±5.69	0.92 (0.91–0.93)	0.51	1.42	2.56 (3.69)	-0.40	
		T3 – T4	69.16±5.69	69.00±6.71	0.89 (0.88–0.90)	0.64	1.80	2.76 (3.98)	0.16	
		Baseline – T4	72.57±6.31	69.00±6.71	0.84 (0.82–0.86)	1.33	3.70	4.71 (6.70)	3.57	
	HR_d2 (in BPM)	Baseline – T1	69.64±13.60	69.83±12.52	0.94 (0.93–0.95)	0.86	2.38	4.95 (7.22)	-0.19	*p* = 0.99,$\eta_p{^2}$=0.0009
		T1 – T2	69.83±12.52	69.64±11.99	0.95 (0.94–0.96)	0.66	1.82	4.16 (5.88)	0.19	
		T2 – T3	69.64±11.99	70.27±13.05	0.96 (0.96–0.97)	0.52	1.43	3.65 (5.17)	-0.64	
		T3 – T4	70.27±13.05	70.59±10.94	0.95 (0.95–0.96)	0.63	1.74	3.97 (5.70)	-0.32	
		Baseline – T4	69.64±13.60	70.59±10.94	0.91 (0.90–0.92)	1.17	3.25	5.52 (8.00)	-0.95	
Boso medicus	SBP_d1 (in mmHg)	Baseline – T1	130.15±12.88	123.83±12.24	0.92 (0.91–0.93)	1.50	4.17	7.61 (6.00)	6.32	*p* < 0.001,$\eta_p{^2}$=0.057
		T1 – T2	123.83±12.24	123.43±11.70	0.94 (0.93–0.94)	0.72	2.01	4.22 (3.46)	0.40	
		T2 – T3	123.43±11.70	122.52±11.54	0.93 (0.92–0.94)	0.84	2.34	4.55 (3.70)	0.91	
		T3 – T4	122.52±11.54	122.34±11.18	0.97 (0.96–0.97)	0.39	1.08	3.22 (2.65)	0.19	
		Baseline – T4	130.15±12.88	122.34±11.18	0.90 (0.89–0.92)	1.97	5.47	8.94 (7.02)	7.82	
	DBP_d1 (in mmHg)	Baseline – T1	80.48±8.15	77.29±7.71	0.91 (0.90–0.92)	0.94	2.61	4.48 (5.75)	3.19	*p* = 0.004,$\eta_p{^2}$=0.05
		T1 – T2	77.29±7.71	75.92±7.40	0.92 (0.91–0.93)	0.67	1.87	3.42 (4.44)	1.37	
		T2 – T3	75.92±7.40	76.02±7.52	0.91 (0.90–0.92)	0.64	1.77	3.05 (3.97)	-0.09	
		T3 – T4	76.02±7.52	76.65±7.01	0.91 (0.90–0.92)	0.61	1.69	2.88 (3.72)	-0.63	
		Baseline – T4	80.48±8.15	76.65±7.01	0.87 (0.85–0.88)	1.24	3.45	4.95 (6.29)	3.83	
	HR_d1 (in BPM)	Baseline – T1	67.65±10.43	65.94±11.08	0.89 (0.88–0.91)	1.17	3.23	4.97 (7.43)	1.71	*p* = 0.872,$\eta_p{^2}$=0.004
		T1 – T2	65.94±11.08	66.42±9.90	0.93 (0.92–0.94)	0.69	1.91	3.70 (5.53)	-0.48	
		T2 – T3	66.42±9.90	67.14±10.82	0.94 (0.93–0.95)	0.54	1.48	3.14 (4.57)	-0.72	
		T3 – T4	67.14±10.82	67.49±10.53	0.94 (0.93–0.95)	0.59	1.63	4.53 (4.99)	-0.35	
		Baseline – T4	67.65±10.43	67.49±10.53	0.92 (0.91–0.93)	0.91	2.52	7.53 (6.75)	0.15	
	SBP_d2 (in mmHg)	Baseline – T1	131.40±13.90	123.71±11.62	0.88 (0.87–0.90)	2.24	6.21	9.00 (7.08)	7.68	*p* < 0.001,$\eta_p{^2}$=0.077
		T1 – T2	123.71±11.62	123.10±11.62	0.94 (0.93–0.95)	0.66	1.84	3.77 (3.11)	0.62	
		T2 – T3	123.10±11.62	123.10±10.71	0.94 (0.93–0.95)	0.64	1.77	3.63 (2.98)	0.00	
		T3 – T4	123.10±10.71	122.60±11.03	0.94 (0.94–0.95)	0.59	1.62	3.32 (2.73)	0.49	
		Baseline – T4	131.40±13.90	122.60±11.03	0.86 (0.85–0.88)	2.55	7.08	9.50 (7.49)	8.79	
	DBP_d2 (in mmHg)	Baseline – T1	79.54±8.62	76.59±8.00	0.90 (0.88–0.91)	0.97	2.68	4.25 (5.52)	2.95	*p* = 0.027,$\eta_p{^2}$=0.04
		T1 – T2	76.59±8.00	76.02±6.91	0.89 (0.88–0.91)	0.80	2.21	3.34 (4.47)	0.57	
		T2 – T3	76.02±6.91	75.68±7.14	0.90 (0.89–0.91)	0.70	1.94	3.08 (4.14)	0.33	
		T3 – T4	75.68±7.14	75.79±7.61	0.86 (0.84–0.87)	0.89	2.48	3.33 (4.40)	-0.11	
		Baseline – T4	79.54±8.62	75.79±7.61	0.79 (0.76–0.81)	1.81	5.02	5.50 (7.05)	3.75	
	HR_d2 (in BPM)	Baseline – T1	68.44±11.43	66.65±10.42	0.93 (0.93–0.94)	0.79	2.19	4.17 (6.47)	1.79	*p* = 0.87,$\eta_p{^2}$=0.004
		T1 – T2	66.65±10.42	67.46±10.28	0.96 (0.95–0.96)	0.47	1.31	3.30 (5.09)	-0.81	
		T2 – T3	67.46±10.28	66.75±9.41	0.96 (0.95–0.96)	0.41	1.11	2.80 (4.15)	0.71	
		T3 – T4	66.75±9.41	67.49±9.33	0.94 (0.93–0.94)	0.61	1.68	3.45 (5.22)	-0.75	
		Baseline – T4	68.44±11.43	67.49±9.33	0.92 (0.91–0.93)	0.95	2.62	4.66 (6.96)	0.95	

Legend: *ANOVA *analysis of variance, *Baseline *arrival measurement without previous standardization, *BPM *beats per minute, *DBP *diastolic blood pressure, *d1 *day 1, *d2 *day 2, *HR *heart rate, *ICC *intraclass correlation coefficient, *M *mean, *MAE *mean absolute error, *MAPE *mean absolute percentage error, *MDC *minimal detectable change, *mean diff. *mean difference, *SBP *systolic blood pressure, *SD *standard deviation, *SEM *Standard error of measurement, *T1 *first blood pressure measurement after 10 min of rest, *T2 *second blood pressure measurement after 10 min of rest with a 3 min rest to T1, *T3 *third blood pressure measurement after 10 min of rest with a 3 min rest to T2, *T4 *fourth blood pressure measurement after 10 min of rest with a 3 min rest to T3, *95% CI *95% confidence interval

#### Welch Allyn ProBP 2000

At baseline, mean systolic/diastolic blood pressure values measured with the Welch Allyn device 117.6±11.5/ 73.0±5.7mmHg on day 1 and 118.6±11.1/72.6±6.3mmHg on day 2. Following the standardized rest period, systolic blood pressure decreased by 4.7 on day 1 and 6.0 mmHg on day 2 from baseline to T1, while diastolic blood decreased by 2.7 and 3.0mmHg, respectively.

Repeated-measures ANOVA revealed significant but small systematic effects across time points for both systolic and diastolic blood pressure (ηp²=0.050–0.058 for systolic, ηp²=0.051 for diastolic; all *p* ≤ 0.003, Table 1). Due to violation of the normal distribution assumption in some parameters, the ANOVA results were confirmed via non-parametric test – without difference in the classification of effects. Post hoc analyses demonstrated that significant differences were primarily confined to comparisons between baseline and later resting measurements (T3 and T4). Specifically, post-hoc tests revealed differences between baseline test and T3 (d = 0.58, *p* = 0.03) and T4 (d = 0.61, *p* = 0.02) on day 1 and on day 2 between baseline and the last two tests (T3: d = 0.68, *p* = 0.007, T4: d = 0.66, *p* = 0.009). The remaining baseline comparisons remained statistically unsignificant (*p* = 0.06–0.19). T1–T4 comparisons were all statistically unsignificant (*p* = 0.83–1.0).

Likewise, for diastolic blood pressure the Scheffé test revealed some significant differences to baseline. On day1, the baseline measurement differed from T2 (d = 0.56, *p* = 0.043) and T3 (d = 0.66, *p* = 0.008), while Baseline to T1 (*p* = 0.17) and T4 (*p* = 0.05) failed in reaching the level of significance (slightly). On day 2, baseline values differed between baseline and T2 (d = 0.60, *p* = 0.026) and T4 (d = 0.56, *p* = 0.045), while the T3 comparison slightly failed in reaching the level of significance (*p* = 0.064). For all post-hoc comparisons, please review Supplemental Table S2.

These findings indicate a systematic reduction in blood pressure values following arrival at the laboratory, while measurements obtained under standardized resting conditions remained stable within sessions when measured with the Welch Allyn device.

#### Boso Medicus vital

Baseline systolic/diastolic blood pressure values measured with the BOSO device were higher compared with the Welch Allyn device, averaging 130.2±12.9/80.5±8.2 mmHg on day 1 and 131.4±13.9/79.5±8.6mmHg on day 2. From baseline to T1, systolic blood pressure decreased by 6.3 mmHg on day 1 and 7.7 mmHg on day 2, with corresponding reductions in diastolic blood pressure of approximately 3 mmHg. The T1 values were 123.8±12.2 (day 1) and 123.7±11.6 mmHg (day2) for diastolic blood pressure. Similarly, diastolic blood pressure decreased from Baseline to T1 with 80.5 ± 8.2 mmHg (day 1) to 77.3±7.7 mmHg and 79.5±8.6 mmHg to 76.6±8.0 mmHg (day 2), respectively. Significant systematic effects across time points were observed for both systolic and diastolic blood pressure (ηp²=0.057–0.077 for systolic, ηp²=0.040–0.050 for diastolic; all *p* ≤ 0.027) (see Table 1). Due to violation of the normal distribution assumption in some parameters, the ANOVA results were confirmed via non-parametric test – without difference in the classification of effects.

Post hoc analyses showed that baseline measurements differed significantly from most resting measurements, whereas no significant differences were detected among T1–T4 measurements. Specifically, on day 1, all baseline comparisons but to T1 (*p* = 0.06) effects were significant in the Scheffé test (d = 0.56–0.66, *p* = 0.008–0.04). No significant effects were observed for T1–T4 comparisons. Also, diastolic blood pressure measurement reached the level of significance for baseline to T2 (d = 0.60, *p* = 0.02) and T3 (d = 0.51, *p* = 0.025), while Baseline to T1 and T4 failed in reaching significant differences (*p* = 0.08–0.22). On day 2, all systolic blood pressure baseline comparisons showed significant systematic changes: Baseline to T1 (d = 0.66, *p* = 0.009), to T2 (d = 0.71, *p* = 0.003), to T3 (d = 0.71, *p* = 0.003) and to T4 (d = 0.76, *p* = 0.001). T1 to T4 comparisons (after the 10 min rest) were all without any significant difference (*p* = 0.90–1.0). For detailed post-hoc test results, please review Table S2 in the Supplemental Material.

Overall, both devices demonstrated a consistent pattern of elevated blood pressure values at baseline followed by stabilization after the standardized rest period.

#### Random errors in systolic and diastolic blood pressure depending on the blood pressure monitor

For both devices, the largest random errors were observed in comparisons involving the baseline measurement. Baseline-to-T1 comparisons yielded MAE values of 6.5–7.0mmHg (5.60%) and 6.95mmHg (6.01%) for systolic and 3.8–4.5mmHg for diastolic blood pressure with the Welch Allyn device, and 7.6–9.0mmHg (systolic) and 4.3–4.5mmHg (diastolic) with the Boso device. Corresponding MAPE values exceeded 6% for systolic blood pressure and 5% for diastolic blood pressure.

In contrast, comparisons among resting measurements (T1–T4) demonstrated substantially smaller random errors. MAE values ranged from 3.2 to 4.7 mmHg (2.8–4.1%) for systolic and 2.2–3.2 mmHg (3.2–4.5%) for diastolic blood pressure for the Welch Allyn device. Comparable values were observed for the BOSO device. Across both devices, baseline-to-rest comparisons consistently produced the highest random error, with MAPE values exceeding 7%, indicating substantial variability when measurements were obtained without adequate rest. Across both devices, baseline to T4 comparisons produced the highest random error, with MAPE values exceeding > 7%, indicating substantial variability when measurements were obtained without adequate rest (Table 1).

#### Relative intraday reliability of both devices

Relative intraday reliability, assessed using ICCs for absolute agreement, was generally high for both devices. ICC values ranged from 0.79 to 0.97 across all intraday comparisons. The lowest reliability estimates were observed in baseline-to-rest comparisons, particularly for diastolic blood pressure measured with the BOSO device (lower 95% confidence interval bound = 0.76). Following the standardized rest period (10 min) ICCs for both systolic and diastolic blood pressure were predominantly (all but the diastolic T3–T4 diastolic measurements) > 0.90, indicating excellent relative reliability. Despite these high ICC values, mean differences between baseline and T1 or T4 condition frequently exceeded the corresponding minimal detectable change (MDC) in both devices, suggesting that systematic changes occurred beyond random measurement error (see Table 1).

### Interday reliability

Interday reliability analyses compared measurements obtained on different days. As no systematic differences were observed among T1 to T4 measurements within sessions, mean values across these four measurements were calculated for interday comparisions.

For both devices, no systematic differences were detected between days for either baseline or resting conditions (*p* = 0.22–0.91). Mean differences between days were consistently well below the MDC for both systolic and diastolic blood pressure. For example, the systolic blood pressure measured via Welch Allyn at baseline was 117.6±11.5 mmHg at day 1 and 118.6±11.1 mmHg at day 2 with a mean difference of -0.86 mmHg (*p* = 0.44).

Random errors across days were slightly larger than intraday random error, with maximum MAPE values reaching approximately 6.7% for diastolic blood pressure measured with Boso device (like intraday comparisons). ICCs values for interday reliability ranged from moderate to excellent. The lowest ICC was observed for diastolic blood pressure measured with the Boso device under baseline conditions (ICC = 0.75; 0.60–0.85, 95%CI), whereas the highest ICC was observed for systolic blood pressure measured with Welch Allyn device (0.92; 0.91–0.93, 95%CI). For more detailed information, please see Table 2.

**Table 2:** Interday reliability metrics comprising descriptives, relative and absolute reliability as well as systematic and random measurement evaluation stratified for device and baseline/rest condition

		Parameter	M±SD	M±SD	ICC (95% CI)	SEM	MDC	MAE (MAPE)	Mean diff.
Welch Allyn	Base	SBP_d1_d2	117.63±11.51	118.60±11.10	0.82 (0.80–0.84)	1.95	5.41	6.51 (5.42)	-0.86 (*p* = 0.44)
		DBP_d1_d2	72.97±5.72	72.57±6.31	0.85 (0.83–0.87)	1.02	2.84	3.42 (4.64)	0.43 (*p* = 0.43)
		HR_d1_d2	71.59±13.12	69.64±13.60	0.75 (0.72–0.78)	2.62	7.27	8.75 (12.36)	2.02 (*p* = 0.19)
	Rest	SBP_d1_d2	111.83±10.03	111.76±9.37	0.92 (0.91–0.93)	1.25	3.46	4.15 (3.66)	0.15 (*p* = 0.83)
		DBP_d1_d2	69.69±5.63	69.14±5.91	0.90 (0.89–0.91)	0.84	2.26	2.71 (5.41)	0.53 (*p* = 0.22)
		HR_d1_d2	70.27±11.17	70.07±11.69	0.80 (0.78–0.83)	2.03	5.64	6.78 (10.77)	0.18 (*p* = 0.88)
Boso medicus	Base	SBP_d1_d2	130.15±12.88	131.40±13.90	0.89 (0.88–0.91)	1.98	5.49	6.60 (6.53)	-0.95 (*p* = 0.37)
		DBP_d1_d2	80.48±8.15	79.54±8.62	0.75 (0.60–0.85)	1.57	4.34	5.22 (7.91)	1.0 (*p* = 0.29)
		HR_d1_d2	67.65±10.43	68.44±11.43	0.72 (0.54–0.82)	2.47	6.85	8.24 (13.44)	-0.87 (*p* = 0.51)
	Rest	SBP_d1_d2	123.03±11.08	123.13±10.53	0.91 (0.90–0.92)	1.42	3.92	4.12 (5.31)	0.09 (*p* = 0.91)
		DBP_d1_d2	76.47±6.93	76.02±6.80	0.78 (0.65–0.87)	1.20	3.34	4.12 (6.70)	0.37 (*p* = 0.62)
		HR_d1_d2	66.75±10.18	67.09±9.48	0.78 (0.65–0.87)	1.84	5.10	6.14 (10.32)	-0.36 (*p* = 0.73)

Legend: *ANOVA *analysis of variance, *Base *arrival measurement without previous standardization, *BPM *beats per minute, *DBP *diastolic blood pressure, *d1 *day 1, *d2 *day 2, *HR *heart rate, *ICC *intraclass correlation coefficient, *M *mean, *MAE *mean absolute error, *MAPE *mean absolute percentage error, *MDC *minimal detectable change, *mean diff. *mean difference, *rest *mean of T1 to T4, *SBP *systolic blood pressure, *SD *standard deviation, *SEM *Standard error of measurement, *95% CI *95% confidence interval

### Device objectivity

Device objectivity was evaluated by comparing blood pressure values obtained with the two oscillometric devices under baseline and resting conditions.

**Fig. 3:**
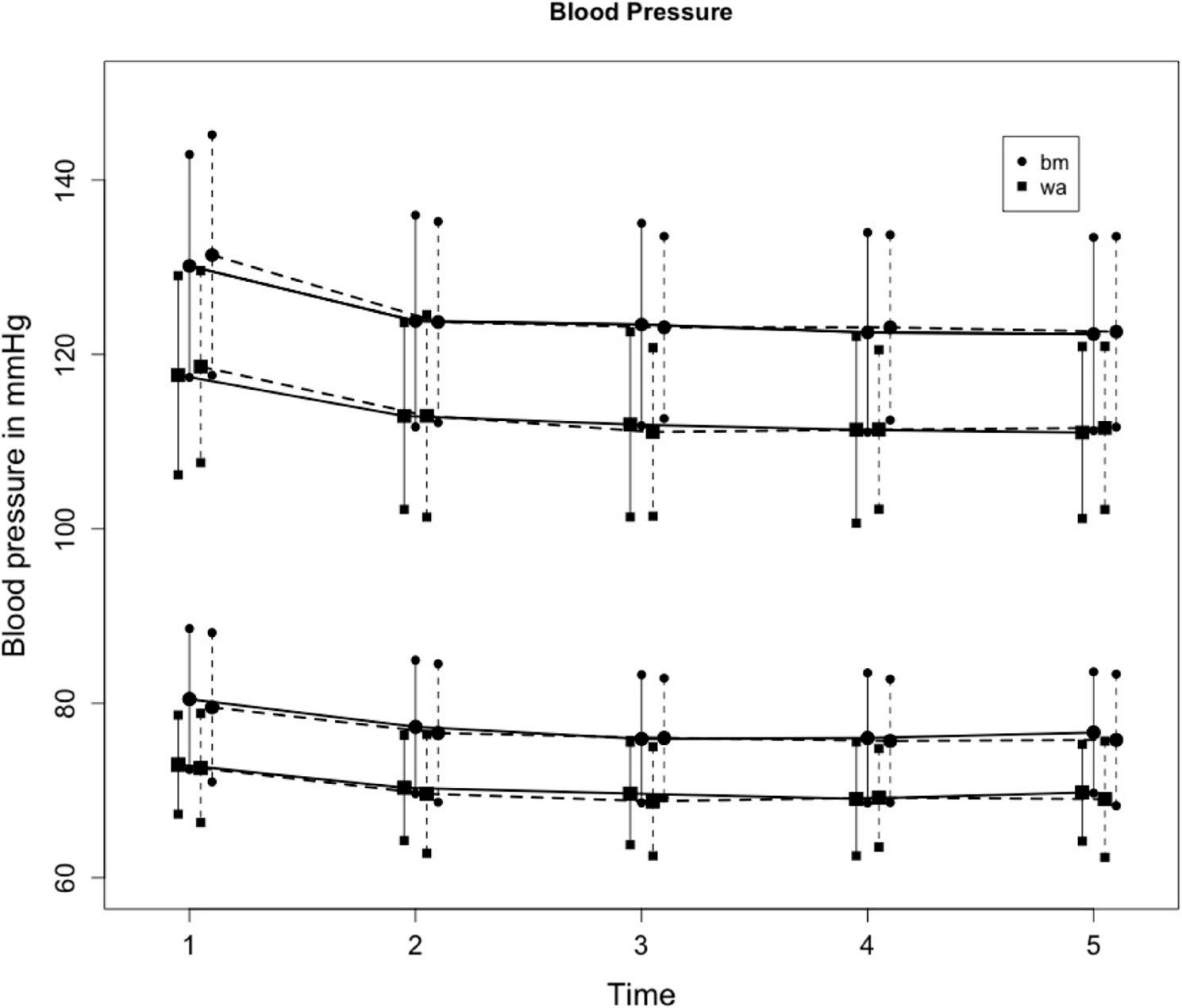
Graphically illustrates the mean and standard deviations for each device on all four testing days and all five time points per day. The permanent lines represent test day 1 the dotted lines stand for test day 2

**Fig. 4:**
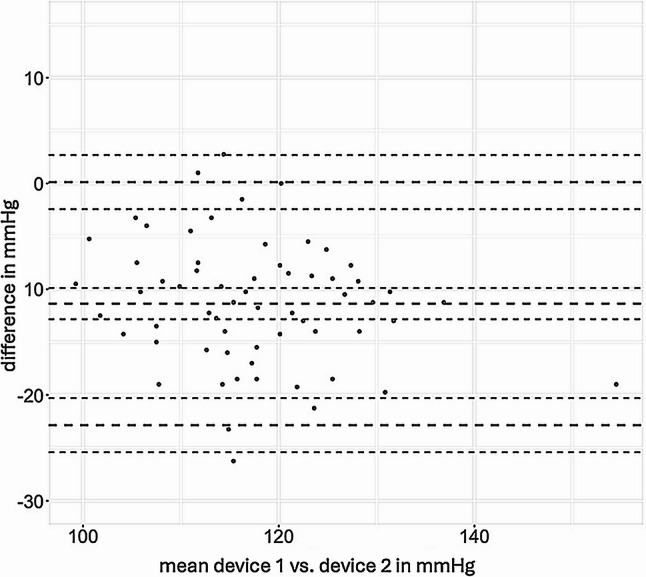
Graphically illustrates the agreement analysis for device objectivity between the Welch Allyn and Boso Medicus device for baseline values

Across all comparisons, a significant systematic bias was observed between devices (all *p* < 0.001). The Welch Allyn device consistently yielded lower systolic and diastolic blood pressure values than the BOSO device. For example, under baseline conditions on day 1, mean systolic blood pressure differed by 12.5mmHg systolic blood pressure and diastolic blood pressure by 7.5mmHg between devices. Similar differences were observed under resting conditions and on day 2 (mean difference: 11.2mmHg in the systolic and 6.8mmHg in the diastolic blood pressure). Mean absolute percentage errors between devices were approximately 10% for both systolic and diastolic blood pressure. In all device comparisons, mean differences exceeded the MDC, indicating clinically meaningful discrepancies. Despite these systematic differences, ICC values ranged from moderate to excellent (ICC = 0.71–0.91), reflecting consistent rank ordering of participants across devices. Device- and day dependent courses of blood pressure measurements are plotted in Fig. 3, while agreement analyses using Bland–Altman plots illustrated wide limits of agreement and confirmed the presence of systematic device-dependent bias (see Fig. 4). For day 2 device comparisons and detailed day 1 comparisons, please review Table 3.

**Table 3:** Comparison between devices (device objectivity) comprising descriptives, relative and absolute reliability as well as systematic and random measurement evaluation stratified for testing day and baseline/rest condition

		Parameter	M±SD	M±SD	ICC (95% CI)	SEM	MDC	MAE (MAPE)	Mean diff.
Day 1	Base	SBP_WA_BM	117.63±11.51	130.15±12.88	0.82 (0.80–0.84)	3.93	10.89	13.09 (10.52)	-12.52 (*p* < 0.001)
		DBP_ WA_BM	72.97±5.72	80.48±8.15	0.71 (0.68–0.75)	1.02	2.84	7.95 (10.21)	-7.51 (*p* < 0.001)
		HR_ WA_BM	71.59±13.12	67.65±10.43	0.65 (0.60–0.69)	2.81	7.80	9.38 (13.39)	3.94 (*p* = 0.011)
	Rest	SBP_ WA_BM	111.83±10.03	123.03±11.08	0.86 (0.85–0.88)	3.43	9.50	11.42 (9.71)	-11.20 (*p* < 0.001)
		DBP_ WA_BM	69.69±5.63	76.47±6.96	0.77 (0.74–0.80)	2.11	5.84	7.03 (9.55)	-6.79 (*p* < 0.001)
		HR_ WA_BM	70.27±11.17	66.75±10.18	0.73 (0.70–0.76)	2.31	6.40	7.70 (10.92)	3.52 (*p* = 0.005)
Day 2	Base	SBP_WA_BM	118.60±11.10	131.40±13.90	0.82 (0.71–0.89)	4.02	11.14	13.19 (10.60)	-12.79 (*p* < 0.001)
		DBP_ WA_BM	72.57±6.31	79.54±8.62	0.80 (0.68–0.88)	2.29	5.35	7.52 (9.89)	-6.97 (*p* < 0.001)
		HR_ WA_BM	69.64±13.60	68.44±11.43	0.77 (0.63–0.86)	2.65	7.35	8.70 (13.04)	1.19 (*p* = 0.39)
	Rest	SBP_ WA_BM	111.76±9.37	123.13±10.53	0.91 (0.89–0.92)	3.45	9.55	11.31 (9.76)	-11.37 (*p* < 0.001)
		DBP_ WA_BM	69.14±5.91	76.02±6.80	0.76 (0.62–0.85)	2.23	6.19	7.32 (10.18)	-6.89 (*p* < 0.001)
		HR_ WA_BM	70.08±11.69	67.09±9.48	0.80 (0.68–0.88)	2.14	5.94	7.03 (10.37)	2.99 (*p* = 0.008)

Legend: *ANOVA *analysis of variance, *Base *arrival measurement without previous standardization, *BM *boso medicus blood pressure device, *BPM *beats per minute, *DBP *diastolic blood pressure, *HR *heart rate, *ICC *intraclass correlation coefficient, *M *mean, *MAE *mean absolute error, *MAPE *mean absolute percentage error, *MDC *minimal detectable change, *mean diff. *mean difference, *rest *mean of T1 to T4, *SBP *systolic blood pressure, *SD *standard deviation, *SEM *Standard error of measurement, *WA *Welch Allyn blood pressure device, *95% CI *95% confidence interval

### Heart rate measurements

Heart rate measurements did not exhibit significant systematic differences across intraday or interday comparisons, nor between devices. Random error for heart rate was comparable to that observed for blood pressure in intraday comparisons but increased in interday and device comparisons, with MAPE values exceeding 13% in some conditions (Table 3).

## Discussion

The present study investigated the reliability, measurement error, and objectivity of oscillometric blood pressure measurements under conditions of high procedural standardization. Three principal findings emerged. First, repeated measurements obtained after an initial rest period demonstrated good to excellent intraday and interday reliability, with intraclass correlation coefficients ranging from 0.79 to 0.97. This level of reliability is in line with previous methodological work indicating that repeated physiological measurements can show high relative stability when assessed under controlled conditions [23, 25]. Second, insufficient rest prior to measurement introduced systematic and random errors of clinically relevant magnitude, with systolic blood pressure values being systematically higher by up to 7.7 mmHg. Third, despite good to excellent intradevice reliability, substantial and consistent systematic differences were observed between two validated oscillometric devices, with mean systolic differences of approximately 11–13 mmHg and diastolic differences of 6–8 mmHg, exceeding the minimal detectable change in all device comparisons.

Together, these findings demonstrate that oscillometric blood pressure measurements may appear internally reliable while still differing meaningfully depending on measurement conditions and device characteristics.

### Reliability versus clinical interpretability

A central finding of this study is the dissociation between high relative reliability and clinical interpretability. Intraclass correlation coefficients indicated good to excellent reliability across most intraday and interday comparisons, particularly after standardized rest. However, as emphasized by Hopkins [24] and Atkinson and Nevill [25], relative reliability metrics such as ICCs primarily describe rank-order consistency and do not quantify the magnitude of measurement error.

This distinction is particularly relevant in blood pressure assessment, where clinical decisions are often based on fixed numerical thresholds. Sakhuja et al. [26] demonstrated that combined systematic and random errors—on the order of 5mmHg systematic bias and a standard deviation of approximately 15 mmHg - may result in hypertension misclassification rates of up to 20%. In the present study, systematic differences between baseline and resting conditions (up to 7.7mmHg) and between devices (up to 13mmHg systolic) frequently exceeded the minimal detectable change, indicating that these differences were unlikely to be attributable to random variation alone [41, 48]. Thus, high relative reliability should not be equated with clinical equivalence or measurement interchangeability.

### Impact of procedural standardization

Measurements obtained immediately after arrival at the laboratory or medical facility were systematically higher and more variable than those obtained after rest, which was expressed in meaningfully enhanced random errors. In the present study, systolic blood pressure decreased by up to 7.7mmHg following rest, a magnitude that falls squarely within the range of rest-related overestimations reported by Kallionen et al. [42] (+ 4.2 to + 11.6mmHg systolic). Similar effects have been described previously by Sala et al. [16] and Nikolic et al. [15], highlighting insufficient rest as a major source of systematic measurement error. In this context, the baseline condition reflected a brief seated period of approximately one minute required for cuff placement, arm circumference assessment, and device preparation, thereby resembling commonly observed clinical practice rather than a completely unstandardized measurement.

Importantly, stabilization of blood pressure did not always occur immediately after the first resting measurement. For the Welch Allyn device, baseline comparisons with T1 and T2 were not consistently significant, whereas comparisons with later measurements (T3 and T4) revealed significant systematic differences. This temporal pattern suggests that even a 10-minute rest period, as commonly recommended in clinical guidelines [45, 50], may be insufficient for full stabilization in some individuals. Nevertheless, once resting conditions were established, no systematic differences were observed among T1–T4 measurements, indicating that standardization substantially improves intraday stability.

### Device-related differences and measurement objectivity

One of the most striking findings of this study was the magnitude and consistency of device-dependent differences. Across baseline and resting conditions, the Welch Allyn device yielded systolic blood pressure values that were on average 11–13 mmHg lower than those obtained with the BOSO device, with corresponding diastolic differences of approximately 6–8 mmHg. These differences exceeded the minimal detectable change in all comparisons and are therefore unlikely to reflect random measurement variability.

Such device-related discrepancies have been reported previously in studies evaluating oscillometric blood pressure measurement accuracy, agreement, and interdevice comparability [51, 52]. Importantly, both devices used in the present study were validated and certified according to established standards [46]. However, as emphasized in previous work, validation does not necessarily imply interchangeability between devices [53].

A plausible explanation for the observed systematic differences lies in device-specific oscillometric measurement techniques and proprietary signal-processing algorithms. Oscillometric devices do not directly measure systolic and diastolic blood pressure but derive these values from cuff pressure oscillations, typically via estimation of mean arterial pressure followed by algorithm-based calculation of systolic and diastolic values [54, 55]. Differences in signal acquisition (e.g., inflation- versus deflation-based measurements) and algorithmic weighting of oscillation amplitudes may therefore lead to consistent over- or underestimation across devices. As these algorithms are not publicly disclosed, their precise impact cannot be directly assessed; nevertheless, the magnitude and consistency of the observed bias strongly suggest a systematic device-related origin rather than random error.

In addition, methodological aspects related to cuff selection may have contributed to the observed interdevice differences. While the Welch Allyn ProBP 2000 allowed cuff-size adaptation according to arm circumference (small adult, adult, and large adult cuffs), the BOSO medicus vital was used with a wide-range standard cuff covering arm circumferences from 22 to 42 cm according to manufacturer specifications. Although all participants were within the recommended cuff range and obesity was excluded, potential influences of cuff design or cuff-size adaptation on interdevice differences cannot be completely excluded.

In this context, a distinction should be made between findings that likely reflect broader characteristics of office oscillometric blood pressure assessment and findings that may be specific to the devices tested in the present study. The effects of insufficient pre-measurement rest, the stabilizing effect of repeated measurements, and the presence of clinically relevant measurement error likely represent broader characteristics of office blood pressure assessment and align with current guideline recommendations. In contrast, the magnitude of the observed interdevice discrepancy should be interpreted more cautiously. As only two validated oscillometric devices with potentially different cuff characteristics and proprietary signal-processing algorithms were examined, the approximately 13 mmHg systolic difference observed here should not be generalized to oscillometric blood pressure monitors as a whole. Nevertheless, both devices represent commonly used validated oscillometric monitors in clinical and ambulatory settings, supporting the practical relevance of the observed findings. Rather, the present findings emphasize that validated devices may still differ systematically and should not automatically be assumed to be interchangeable in routine clinical practice.

### Clinical implications

From a clinical perspective, the findings of this study have important implications. First, reliance on single blood pressure measurements—particularly those obtained without adequate rest—may substantially increase the risk of misclassification, as previously emphasized by Sakhuja et al. [26] and Kallionen et al. [42]. In the present study, baseline measurements differed from resting values by up to 7–8 mmHg, a magnitude that alone may shift individuals across diagnostic thresholds.

Second, device-dependent differences of approximately 13 mmHg in systolic blood pressure may meaningfully influence blood pressure classification under contemporary guideline thresholds [5, 8]. However, current hypertension guidelines already recommend repeated office measurements and, whenever feasible, confirmation through home or ambulatory blood pressure monitoring prior to treatment decisions [43]. Nevertheless, review studies have identified persistent knowledge gaps among healthcare professionals regarding guideline-recommended and standardized blood pressure measurement procedures [33]. In this context, the present findings primarily emphasize the importance of standardized procedures and cautious interpretation of isolated office blood pressure readings, particularly when values are close to diagnostic thresholds.

These findings support recommendations advocating repeated measurements, strict adherence to standardized protocols, and cautious interpretation of isolated blood pressure values [27, 45]. Where ambulatory blood pressure monitoring is unavailable, repeated office or home measurements should be interpreted as ranges rather than precise point estimates, particularly when values are close to diagnostic cut-offs.

Importantly, the present findings should not be interpreted as questioning the utility of office oscillometric blood pressure assessment, which remains a cornerstone of hypertension screening and management according to contemporary guidelines. Rather, the findings reinforce current recommendations advocating repeated office measurements and, where feasible, confirmation through home or ambulatory blood pressure monitoring, particularly when values are close to diagnostic thresholds.

### Limitations

Several limitations should be acknowledged. First of all, no gold standard measurement (e.g. intra-arterial measurement, validated mercury sphygmomanometer [56], expert auscultatory measurement [57]) was performed, making conclusions about if one device (and if one, which one) measured the blood pressure accurately. Accordingly, future studies should evaluate the validity of both devices in healthy participants, at from this investigation it could only be concluded that there is a clinically discrepancy between the devices. Furthermore, measurements were exclusively conducted in comparatively young and healthy adults without diagnosed hypertension or other diseases that can influence the resting blood pressure (e.g., diabetes [58, 59] or obesity [60, 61]. Similarly, the four laboratory visits do not fully reflect typical clinical blood pressure assessment or the complexity of real-world hypertension diagnosis, which should be considered when interpreting the findings.

Simultaneous measurement with both devices on the same arm was not feasible. Although the device order was randomized, arm-related effects other time related effects cannot be fully excluded [42]. Furthermore, the true underlying blood pressure of participants remains unknown, preventing conclusions regarding absolute over- or underestimation by either device. Some measurements deviated from normality assumptions; however, non-parametric sensitivity analyses yielded comparable results. Furthermore, although all devices were used according to manufacturer recommendations, differences in cuff design and cuff-size adaptation between devices may have contributed to the observed interdevice differences and cannot be fully excluded.

Finally, the proprietary nature of oscillometric algorithms limits mechanistic interpretation of device-related differences, although the systematic nature of the observed bias argues against random variability as the primary explanation.

### Conclusions

Under conditions of high procedural standardization, oscillometric blood pressure measurements demonstrate good to excellent reliability within and across sessions. However, insufficient rest and device-specific measurement characteristics introduce systematic and random errors of clinically relevant magnitude. In the present study, device-dependent differences of approximately 13 mmHg in systolic blood pressure and baseline-to-rest differences of up to 8 mmHg highlight that high relative reliability does not ensure clinical equivalence. Measurement reliability, standardization, and device characteristics must therefore be considered to avoid misclassification and inappropriate clinical decision-making.

## Supplementary Information

Supplementary Material 1.

## Data Availability

Original data can be requested from the corresponding author due to reasonable request.

## Abbreviations

mmHG: Millimetres of mercury
ICC: Intraclass correlation coefficient
cm: Centimeters
kg: Kilogram
kg/m2: Kilograms per square meter
ESH: European Society of Hypertension
M: Means
SD: Standard deviations
ANOVA: Analyses of variance
ηp²: Partial eta squared
d: Cohen’s d
MSR: Mean square of rows
MSE: Mean square error
MSC: Mean square of columns
CI: Confidence interval
SEM: Standard error of measurement
MDC: Minimal detectable change
MAE: Mean absolute error
MAPE: Mean absolute percentage error
