# Supplementary material for: Reliability and device objectivity in oscillatory blood pressure measurement – a measurement error analysis to inform clinical decision making
Source: BMC Cardiovasc Disord. 2026 Jul 14;26:593. doi: 10.1186/s12872-026-06262-1 (PMC13366944; doi:10.1186/s12872-026-06262-1)
Supplement: Supplementary file 1 — Supplementary Material 1. [file 12872_2026_6262_MOESM1_ESM.docx]

**SUPPLEMENTAL MATERIAL**

**Table S1** Descriptive Statistics for Median and 25% and 75% Percentile reporting with Shapiro Wilk p-value check

|  |  | **Median** | **25 – 75% Percentiles** | **Shapiro Wilk p-value** |
| --- | --- | --- | --- | --- |
| **Day 1** | **Boso Medicus SBP** | 129.00 | 120.00 – 136.00 | 0.240 |
|  |  | 124.00 | 115.00 – 131.00 | 0.008 |
|  |  | 124.00 | 116.00 – 129.00 | 0.195 |
|  |  | 122.00 | 116.00 – 128.00 | <0.001 |
|  |  | 120.00 | 115.00 – 128.00 | <0.001 |
|  | **Boso Medicus DBP** | 80.00 | 75.00 – 85.00 | 0.015 |
|  |  | 76.00 | 72.00 – 82.00 | <0.001 |
|  |  | 76.00 | 71.00 – 81.00 | 0.017 |
|  |  | 75.00 | 70.00 – 80.00 | <0.001 |
|  |  | 76.00 | 72.00 – 81.00 | 0.060 |
|  | **Welch Allyn SBP** | 115.00 | 109.00 – 124.00 | 0.005 |
|  |  | 111.00 | 104.00 – 120.00 | 0.004 |
|  |  | 109.00 | 104.00 – 120.00 | <0.001 |
|  |  | 110.00 | 103.00 – 116.00 | <0.001 |
|  |  | 110.00 | 105.00 – 115.00 | <0.001 |
|  | **Welch Allyn DBP** | 73.00 | 69.00 – 76.00 | 0.028 |
|  |  | 69.00 | 66.00 – 73.00 | 0.020 |
|  |  | 68.00 | 66.00 – 74.00 | 0.004 |
|  |  | 68.00 | 65.00 – 71.00 | <0.001 |
|  |  | 69.00 | 66.00 – 73.00 | 0.085 |
| **Day 2** | **Boso Medicus SBP** | 130.50 | 121.00 – 140.00 | 0.258 |
|  |  | 124.00 | 117.00 – 131.00 | 0.017 |
|  |  | 124.00 | 115.00 – 129.00 | 0.013 |
|  |  | 123.00 | 116.00 – 130.00 | 0.206 |
|  |  | 122.00 | 115.50 – 128.00 | 0.024 |
|  | **Boso Medicus DBP** | 78.00 | 74.50 – 82.00 | <0.001 |
|  |  | 77.00 | 72.00 – 80.00 | 0.085 |
|  |  | 76.00 | 71.00 – 79.00 | 0.025 |
|  |  | 75.00 | 71.00 – 80.00 | 0.344 |
|  |  | 74.00 | 70.00 – 80.00 | <0.001 |
|  | **Welch Allyn SBP** | 119.00 | 110.00 – 126.50 | 0.458 |
|  |  | 112.00 | 104.50 – 120.50 | 0.063 |
|  |  | 110.00 | 103.00 – 119.50 | 0.176 |
|  |  | 111.00 | 105.50 – 117.50 | 0.118 |
|  |  | 112.00 | 105.00 – 116.50 | 0.054 |
|  | **Welch Allyn DBP** | 72.00 | 68.00 – 75.50 | 0.032 |
|  |  | 69.00 | 65.00 – 73.00 | 0.077 |
|  |  | 68.00 | 64.00 – 73.50 | 0.324 |
|  |  | 68.00 | 65.00 – 73.00 | 0.124 |
|  |  | 69.00 | 64.00 – 73.50 | 0.174 |

*Legend: DBP = diastolic blood pressure, SBP = systolic blood pressure*

**Table S2** Scheffé post hoc test overview table stratified for testing day and measurement device

|  |  | **Comparison** | **Mean difference** | **Cohens_d** | **p-value** |  |  | **Comparison** | **Mean difference** | **Cohens_d** | **p-value** |
| --- | --- | --- | --- | --- | --- | --- | --- | --- | --- | --- | --- |
| **Day 1** | **BM_SBP** | Baseline _ T1 | 6.32 | 0.53 | 0.060* | **Day 2** | **BM_SBP** | Baseline _ T1 | 7.46 | 0.64 | 0.012 |
|  |  | Baseline _ T2 | 6.72 | 0.56 | 0.037 |  |  | Baseline _ T2 | 8.03 | 0.69 | 0.005 |
|  |  | Baseline _ T3 | 7.63 | 0.64 | 0.011 |  |  | Baseline _ T3 | 8.03 | 0.69 | 0.005 |
|  |  | Baseline _ T4 | 7.82 | 0.66 | 0.008 |  |  | Baseline _ T4 | 8.52 | 0.73 | 0.002 |
|  |  | T1_ T2 | 0.40 | 0.03 | 1.000 |  |  | T1_ T2 | 0.57 | 0.05 | 0.999 |
|  |  | T1_ T3 | 1.31 | 0.11 | 0.983 |  |  | T1_ T3 | 0.57 | 0.05 | 0.999 |
|  |  | T1_ T4 | 1.49 | 0.13 | 0.972 |  |  | T1_ T4 | 1.06 | 0.09 | 0.992 |
|  |  | T2_ T3 | 0.91 | 0.08 | 0.996 |  |  | T2_ T3 | 0.00 | 0.00 | 1.000 |
|  |  | T2_ T4 | 1.09 | 0.09 | 0.991 |  |  | T2_ T4 | 0.49 | 0.04 | 1.000 |
|  |  | T3_ T4 | 0.19 | 0.015 | 1.00 |  |  | T3_ T4 | 0.49 | 0.04 | 1.000 |
|  | **BM_DBP** | Baseline _ T1 | 3.19 | 0.42 | 0.221 |  | **BM_DBP** | Baseline _ T1 | 2.95 | 0.38 | 0.327 |
|  |  | Baseline _ T2 | 4.55 | 0.60 | 0.021 |  |  | Baseline _ T2 | 3.52 | 0.46 | 0.160 |
|  |  | Baseline _ T3 | 4.46 | 0.59 | 0.025 |  |  | Baseline _ T3 | 3.86 | 0.50 | 0.096 |
|  |  | Baseline _ T4 | 3.83 | 0.51 | 0.083 |  |  | Baseline _ T4 | 3.75 | 0.49 | 0.115* |
|  |  | T1_ T2 | 1.37 | 0.18 | 0.900 |  |  | T1_ T2 | 0.57 | 0.07 | 0.996 |
|  |  | T1_ T3 | 1.27 | 0.17 | 0.921 |  |  | T1_ T3 | 0.91 | 0.12 | 0.979 |
|  |  | T1_ T4 | 0.65 | 0.09 | 0.993 |  |  | T1_ T4 | 0.79 | 0.10 | 0.987 |
|  |  | T2_ T3 | -0.09 | -0.01 | 1.000 |  |  | T2_ T3 | 0.33 | 0.04 | 1.000 |
|  |  | T2_ T4 | -0.72 | -0.55 | 0.990 |  |  | T2_ T4 | 0.22 | 0.03 | 1.000 |
|  |  | T3_ T4 | -0.63 | -0.48 | 0.994 |  |  | T3_ T4 | -0.11 | -0.01 | 1.000 |
|  | **WA_SBP** | Baseline _ T1 | 4.69 | 0.44 | 0.188 |  | **WA_SBP** | Baseline _ T1 | 5.65 | 0.55 | 0.052* |
|  |  | Baseline _ T2 | 5.66 | 0.53 | 0.063* |  |  | Baseline _ T2 | 7.49 | 0.73 | 0.003 |
|  |  | Baseline _ T3 | 6.26 | 0.58 | 0.028 |  |  | Baseline _ T3 | 7.21 | 0.70 | 0.004 |
|  |  | Baseline _ T4 | 6.58 | 0.61 | 0.017 |  |  | Baseline _ T4 | 7.03 | 0.68 | 0.006* |
|  |  | T1_ T2 | 0.97 | 0.09 | 0.992 |  |  | T1_ T2 | 1.84 | 0.18 | 0.908 |
|  |  | T1_ T3 | 1.57 | 0.15 | 0.952 |  |  | T1_ T3 | 1.56 | 0.15 | 0.949 |
|  |  | T1_ T4 | 1.89 | 0.18 | 0.909 |  |  | T1_ T4 | 1.38 | 0.13 | 0.966 |
|  |  | T2_ T3 | 0.60 | 0.06 | 0.999 |  |  | T2_ T3 | -0.29 | -0.03 | 1.000 |
|  |  | T2_ T4 | 0.92 | 0.09 | 0.993 |  |  | T2_ T4 | -0.46 | -0.05 | 1.000 |
|  |  | T3_ T4 | 0.32 | 0.03 | 1.000 |  |  | T3_ T4 | -0.18 | -0.02 | 1.000 |
|  | **WA_DBP** | Baseline _ T1 | 2.66 | 0.44 | 0.173* |  | **WA_DBP** | Baseline _ T1 | 2.95 | 0.46 | 0.154 |
|  |  | Baseline _ T2 | 3.32 | 0.56 | 0.043 |  |  | Baseline _ T2 | 3.81 | 0.60 | 0.026 |
|  |  | Baseline _ T3 | 3.94 | 0.66 | 0.008 |  |  | Baseline _ T3 | 3.41 | 0.53 | 0.064 |
|  |  | Baseline _ T4 | 3.22 | 0.54 | 0.055* |  |  | Baseline _ T4 | 3.57 | 0.56 | 0.045 |
|  |  | T1_ T2 | 0.66 | 0.11 | 0.983 |  |  | T1_ T2 | 0.86 | 0.13 | 0.967 |
|  |  | T1_ T3 | 1.28 | 0.21 | 0.831 |  |  | T1_ T3 | 0.46 | 0.07 | 0.997 |
|  |  | T1_ T4 | 0.55 | 0.09 | 0.991 |  |  | T1_ T4 | 0.62 | 0.10 | 0.990 |
|  |  | T2_ T3 | 0.62 | 0.10 | 0.987 |  |  | T2_ T3 | -0.40 | -0.06 | 0.998 |
|  |  | T2_ T4 | -0.11 | -0.02 | 1.000 |  |  | T2_ T4 | -0.24 | -0.04 | 1.000 |
|  |  | T3_ T4 | -0.72 | -0.12 | 0.976 |  |  | T3_ T4 | 0.16 | 0.03 | 1.000 |

Legend: *Baseline = arrival measurement without previous standardization,* BM = Boso Medicus blood pressure monitor, DBP = diastolic blood pressure, *T1 = first blood pressure measurement after 10 minutes of rest, T2 = second blood pressure measurement after 10 minutes of rest with a 3 minutes rest to T1, T3 = third blood pressure measurement after 10 minutes of rest with a 3 minutes rest to T2, T4 = fourth blood pressure measurement after 10 minutes of rest with a 3 minutes rest to T3, WA = Welch Allyn blood pressure monitor, * = reaching level of significance when using the Dunn’s Post-Hoc Test for non-parametric tests*
